# Supplementary material for: Dynamic allele usage of X-linked genes ameliorates neurodevelopmental disease phenotypes in brain organoids
Source: Nat Commun. 2026 Jan 14;17:599. doi: 10.1038/s41467-026-68428-x (PMC12808108; doi:10.1038/s41467-026-68428-x)

Supplemental information

Supplementary Figures and Figure legends

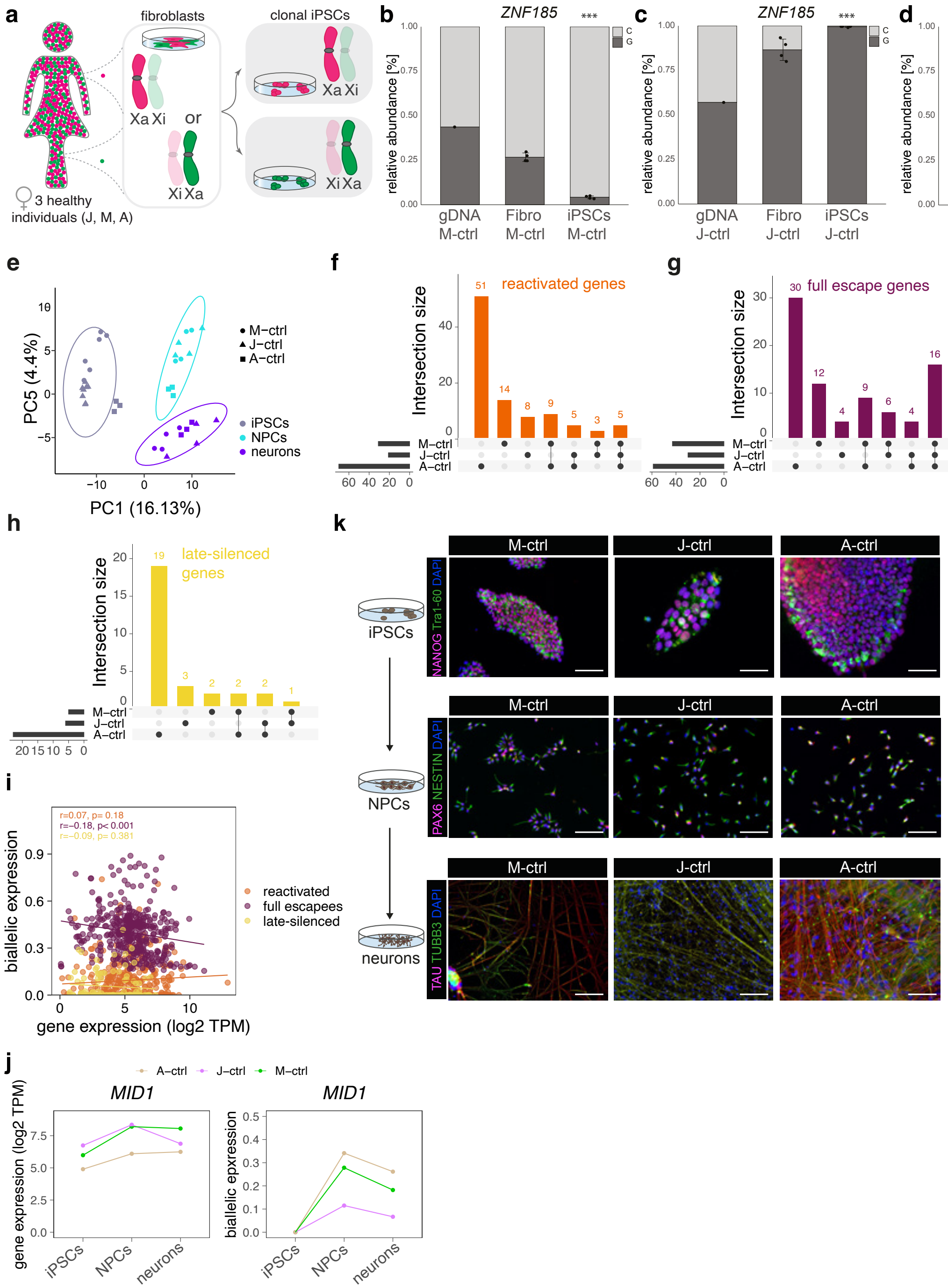

Fig. S1. Characterization of X-reactivation. a, Scheme depicting the logic of iPSC clone selection carrying heterozygous sequence variants in X-chromosomal genes, producing distinct iPSC clones with one (pink) or the other (green) X-chromosome active. **b-d**, Relative allele-

specific expression of *ZNF185* transcripts in M-line (**b**), J-line (**c**), and A-line cells (**d**) analyzed by QUASEP. In each panel, the small grey boxes labelled with a single letter (C, G) indicate the DNA base present at each of the two alleles at the heterozygous SNP used in the analysis. *P*-values: **b**, P $<1.0\text{x}10^{-4}$; **c**, $P = 7.9\text{x}10^{-4}$; **d**, $P = 0.0362$. Sample sizes: **b**, gDNA n=1, all other samples *n*=4; **c**, gDNA n=1, all other samples *n*=4; **d**, gDNA n=1, all other samples *n*=2. Testing for statistical significance was performed for the values in iPSCs compared to the values in the corresponding fibroblasts. One-way ANOVA followed by Tukey's multiple comparisons test; values are mean ± SD; **P*<0.05, ***P*<0.01, ****P*<0.001. **e,** Principal component analysis of allelic ratios (number of reads mapping to the reference allele divided by the total number of reads) based on X-chromosomal variant sites including 95% confidence ellipses confirmed a unique X-chromosomal allele-specific expression profile for iPSCs, NPCs and neurons in the M-ctrl, J-ctrl, and A-ctrl cell lines. **f,** UpSet plot indicating the number of genes biallelically expressed in NPCs and/or neurons but not in iPSCs (reactivated genes) in the three different donor lines and their intersections. **g,** UpSet plot with the number of genes biallelically expressed in iPSCs, NPCs and neurons (full escapees) in the three different donor lines and their intersections. **h,** UpSet plot indicating the number of genes biallelically expressed in iPSCs only (late-silenced genes) in the three different donor lines and their intersections. **i,** Scatter plot showing biallelic expression (Xi/total allelic expression) versus total gene expression for all reactivated, full escape, and late-silenced genes. Regression analysis reveals no significant correlation between gene expression levels and biallelic expression for reactivated and late-silenced genes. **j,** Line plots showing the overall gene expression (left) and biallelic expression (right) for *MID1* at different differentiation stages in the three cell lines A, M and J. Overall expression is shown as log2 transcripts per million (TPM) counts and biallelic expression corresponds to the allelic ratio of reads mapping to Xi to the sum of reads mapping to Xi and Xa. Xi: inactive X-chromosome, Xa: active X-chromosome. **k,** Images showing immunohistochemical stainings of iPSCs (upper panel), NPCs (middle panel), and neurons (lower panel) highlighting positivity for the respective markers (indicated on the left) across the M-ctrl (left), the J-ctrl (middle), and the A-ctrl (right) lines. Scale bar = 130 µm. Scale bar = 70 µm for A-ctrl neurons.

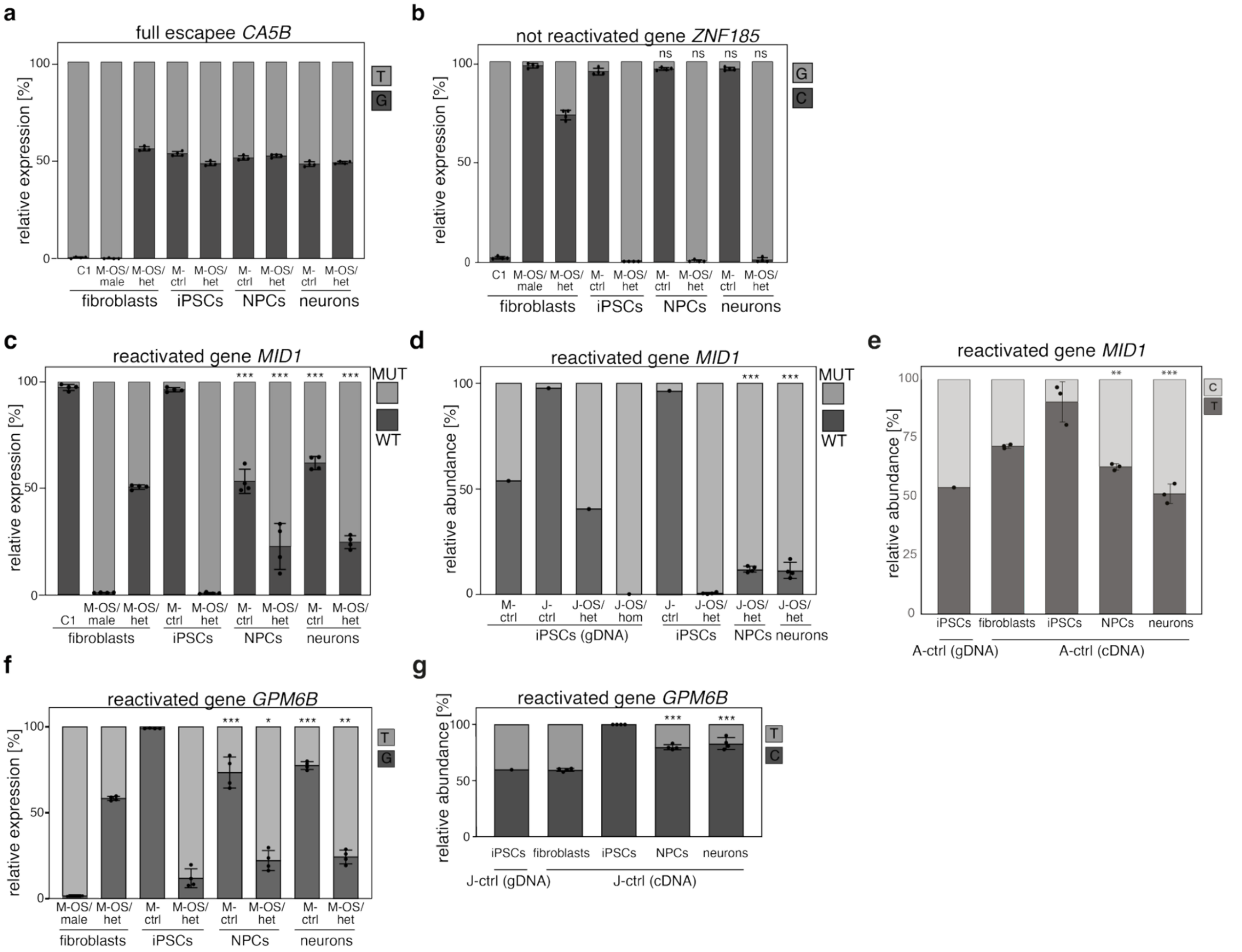

Fig. S2. Characterization of X-reactivation and X-reactivated genes using QUASEP. a, Relative allele-specific expression of *CA5B* transcripts in M-ctrl and M-OS/het fibroblasts, iPSCs, NPCs, and neurons analyzed by QUASEP. **b,** Relative allele-specific expression of *ZNF185* transcripts in M-ctrl and M-OS/het fibroblasts, iPSCs, NPCs, and neurons analyzed by using QUASEP. Note that *ZNF185* does not show biallelic expression in iPSCs, NPCs or neurons (exact *P*-value from left to right: 0.81, >0.99, 0.80, 0.99). **c,** Relative expression of mutant and wildtype *MID1* transcript in M-ctrl and M-OS/het fibroblasts, iPSCs, NPCs, and neurons analyzed by QUASEP (*P*-values from left to right: $<1.0x10^{-4}$, $<1.0x10^{-4}$, $<1.0x10^{-4}$, $<1.0x10^{-4}$). **d,** Relative abundance of mutant and wildtype *MID1* gene and transcript in M-ctrl, J-ctrl, J-OS/het and J-OS/hom iPSCs, NPCs, and neurons analyzed by QUASEP (*P*-values from left to right: $2.0x10^{-4}$, $2.0x10^{-4}$). **e,** QUASEP analysis of the relative abundance of *MID1* gene and transcript in A-ctrl genomic DNA, fibroblasts, iPSCs, NPCs, and neurons (*P*-values from left to right: 0.022, $3x10^{-4}$)**.** **f,** Relative allele-specific expression of *GPM6B* in M-ctrl and M-OS/het fibroblasts, iPSCs, NPCs,

and neurons analyzed by QUASEP (*P*-values from left to right: <1.0x10^{-4}, 1.6x10^{-2}, <1.0x10^{-4}, 4.2x10^{-3}). **g,** QUASEP analysis of the relative abundance of *GPM6B* gene and transcript in J-ctrl fibroblasts, iPSCs, NPCs, and neurons (*P*-values from left to right: <1.0x10^{-4}, <1.0x10^{-4}). **a-g,** the unrelated wildtype male control line C1 and the hemizygous mutant M-OS/male fibroblasts are used as controls. In each panel, the small grey boxes labeled with a single letter (T, C, G) or with MUT and WT indicate the DNA base or the patient-specific mutation present at each of the two alleles at the heterozygous SNP site used in the analysis. Dots represent independent samples; all iPSC (gDNA), J-ctrl iPSC cDNA n=1; A-ctrl fibroblasts, iPSCs, NPCs, and neurons *n*=3; all other samples *n*=4; testing for statistical significance was performed for the values in NPCs and neurons compared to the values in the corresponding iPSCs. One-way ANOVA followed by Tukey's multiple comparisons test; values are mean ± SD; **P*<0.05, ***P*<0.01, ****P*<0.001, ns *P*>0.05.

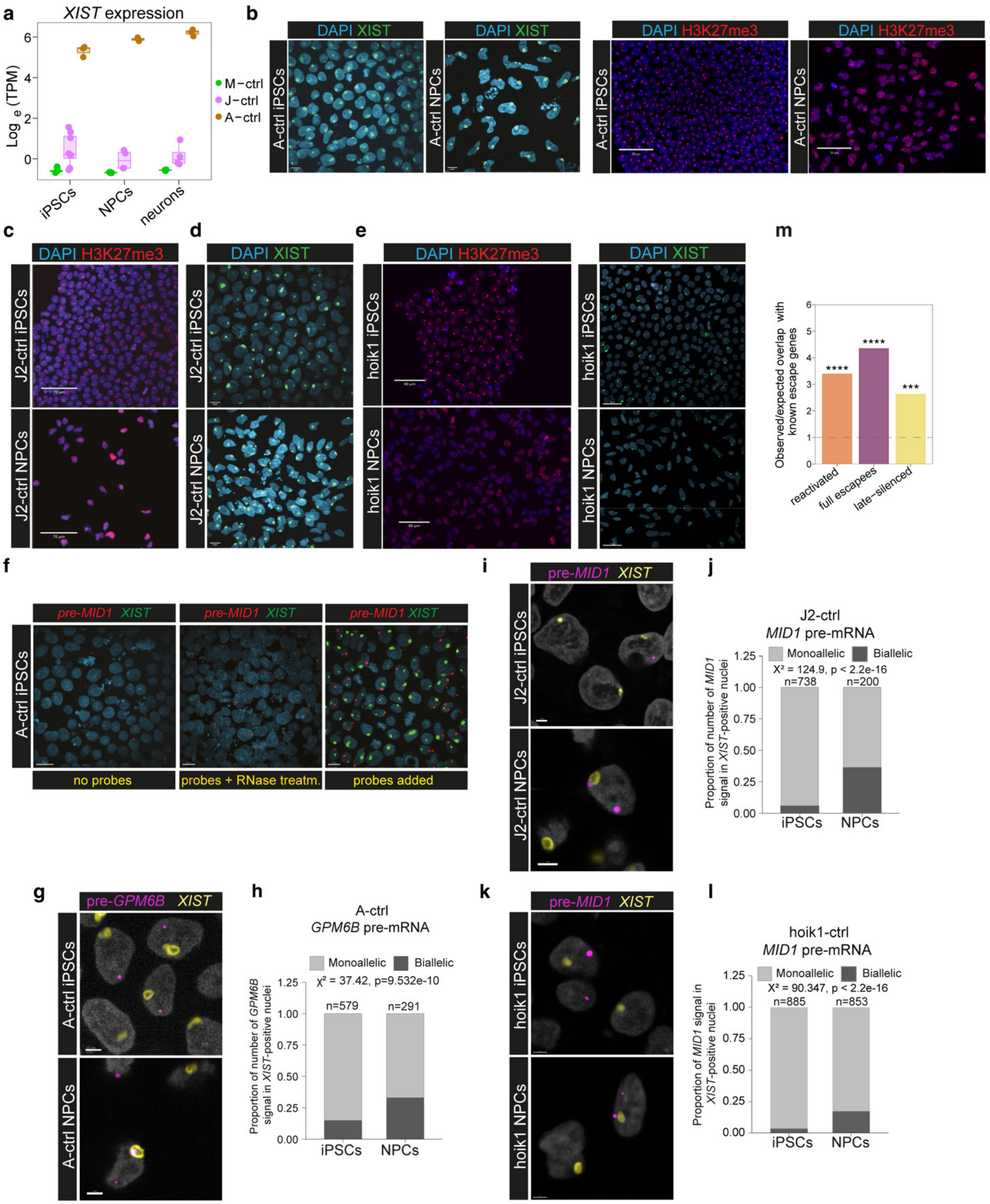

Fig. S3. Single nucleus evaluation of biallelic expression in *XIST* high and low iPSC control lines. a, Boxplots showing the *XIST* expression levels in iPSCs, NPCs and neurons of the M-ctrl, J-ctrl and A-ctrl donor lines. Values are shown as $\log_e$-transformed transcripts per million (TPM).

M-ctrl iPSCs: *n*=6; J-ctrl iPSCs:*n*=8; M-ctrl NPCs: *n*=4; J-ctrl NPCs: *n*=4; M-ctrl neurons: *n*=4; J-ctrl neurons: *n*=4; A-ctrl iPSCs, NPCs and neurons: *n*=3. Boxplots show median, quartiles (box), and range (whiskers). **b,** Maximum intensity projection showing expression of XIST (green) in nuclei of iPSCs and NPCs (left panel) as well as H3K27me3 (red signal) immunofluorescence of iPSC and NPCs (right panel) of the A-ctrl line. Scale bars = 9µm and 70 µm, respectively. **c,** Immunofluorescence stainings of J2-ctrl line cells showing H3K27me3 foci (red) in nuclei (blue) in iPSCs (upper panel) and NPCs (lower panel). Scale bar = 70 µm. **d,** Maximum intensity projection showing the presence of *XIST* (green) in DAPI-stained nuclei (blue) of the *XIST* expressing J2-ctrl iPSCs (upper panel) and NPCs (lower panel). Scale bar = 9µm. **e,** Immunofluorescence of H3K27me3 foci (red) in iPSCs and NPCs (left panel) as well as single focal plane image from a Z-stack of an RNA-FISH experiment showing the *XIST* (green) in iPSCs and NPCs from the *XIST*-expressing hoik1-ctrl line. Scale bars = 60µm and 3µm, respectively. **f,** Maximum intensity projections showing the technical controls for the RNA-FISH experiments in Figs. 1, 3, and S3. For all probes, two controls were performed, i.e. addition of no probe, but same experimental procedure (left panel) as well as RNase treatment prior to the addition of probes (middle panel). Right panel shows a representative experiment using the probes against *XIST* and pre-mRNA of *MID1*. Scale bars =10µm and scale bar =15µm for no probes. All experiments were conducted using samples with at least 70% of *XIST*-positive cells. **g,** Representative single focal plane image from a Z-stack of RNA-FISH targeting *XIST* (yellow) and the pre-mRNA of the reactivated gene *GPM6B* (magenta) in iPSCs (upper panel) and NPCs (lower panel) from the A-ctrl line. Scale bar = 3µm. **h,** Quantification of mono- and biallelic nuclear *GPM6B* signal in *XIST*-positive nuclei in the A-ctrl line. Biallelic *GPM6B* in iPSCs: 0.153; NPCs: 0.324. *n* = individual cell nuclei, indicated above the bars. Statistical comparisons were facilitated with a Chi-squared test. All experiments were conducted using samples with at least 70% of *XIST*-positive cells. **i,** Representative image of a single focal plane image of an RNA-FISH experiment against *XIST* (yellow) and the pre-mRNA of *MID1* (magenta) in iPSCs (upper panel) and NPCs (lower panel) from the *XIST*-expressing J2-ctrl line. Scale bar = 3µm. **j,** Quantification of mono- and biallelic nuclear *MID1* signal in *XIST*-positive nuclei in the J2-ctrl line. Biallelic *MID1* in iPSCs: 0.058; NPCs: 0.337. **k,** Representative image of a single focal plane image of an RNA-FISH experiment against *XIST* (yellow) and the pre-mRNA of *MID1* (magenta) in iPSCs (upper panel) and NPCs (lower panel) from the *XIST*-expressing hoik1-ctrl line. Scale bar = 3µm. **l,** Quantification of

mono- and biallelic nuclear *MID1* signal in *XIST*-positive nuclei in the hoik1-ctrl line. Biallelic *MID1* in iPSCs: 0.032; NPCs: 0.171. For **h, j, l**, *n* = individual cell nuclei, indicated above the bars. Statistical comparison was performed using a Chi-squared test for association. **m,** Bar plot showing the ratio of the observed to expected overlap of reactivated, full escape and late-silenced genes with previously reported escape genes in humans. Exact *P*-values (left to right): 2.2*10-16, 2.2*10-16, 0.00038. *P*-values were calculated using the cumulative distribution function of the normal distribution.

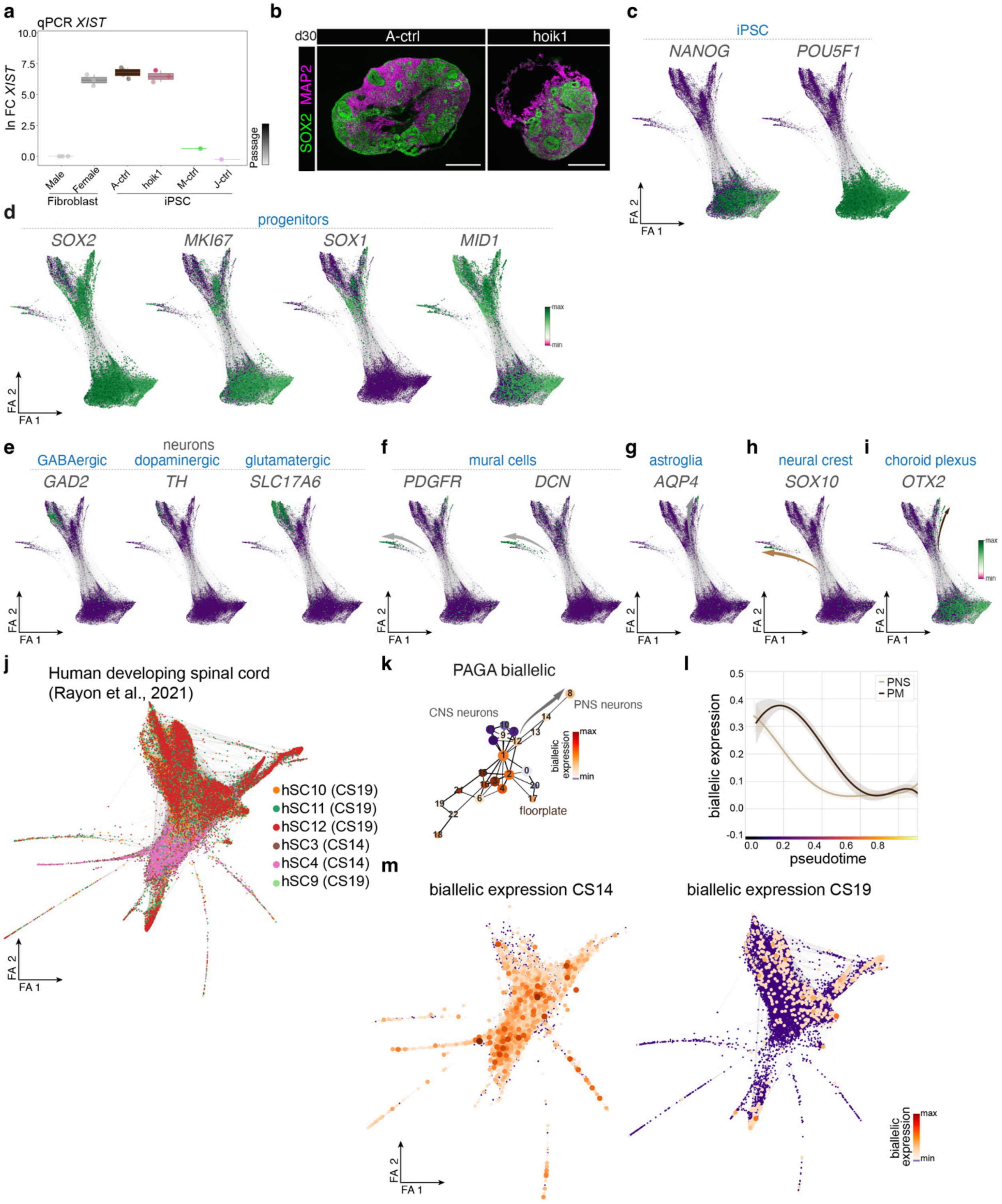

Fig. S4. Allele-specific analysis of X-linked genes in brain organoids and in the developing human spinal cord. a, Quantitative RT-PCR data for the detection of *XIST* expression across male and female primary fibroblasts and different iPSC lines confirming high *XIST* levels in the A-ctrl

and the hoik1 line. Boxplots show median, quartiles (box), and range (whiskers). Dots represent technical replicates for fibroblasts, and samples of independent cultures in iPSCs. **b,** Images showing representative brain organoid slices stained for the progenitor marker SOX2 (green) and the neuronal marker MAP2 (magenta) on d30. Scale bar = 500µm. **c-i,** Molecular characterization of cell types and lineages contained in the snRNA-seq data set including iPSCs and organoids (d30) of the A-ctrl and the hoik1 line. Expression of feature genes is projected onto the force-directed graph embedding shown in Fig. 4a. Pluripotency genes marking iPSCs (**c**), genes expressed in neural progenitors (**d**), presence of distinct neuronal populations shown by expression of *GAD2*, *TH*, and *SLC17A6* (**e**), mural cells (**f**), astroglia (**g**), cells of neural crest origin (**h**), and choroid plexus identity (**i**). **j,** Force-directed graph embedding showing annotations of the six female samples deduced from the Rayon et al. dataset of the developing human spinal cord [28]. CS = carnegie stage. **k,** Degree of biallelic expression of non-pseudoautosomal genes shown in PAGA graph focusing on distinct clusters (as shown in Fig. 4g). **l,** Fitted line plot showing biallelic expression of non-pseudoautosomal genes in PM (paraxial mesoderm, clusters 3, 4, 6) and PNS (peripheral nerve system, clusters 8, 13, 14) lineages along pseudotime. 95% confidence interval is shown as a transparent band in the same respective color. **m,** Degree of biallelic expression of non-pseudautosomal genes separated by CS14 and CS19. In **c-j, m** FA refers to force atlas.

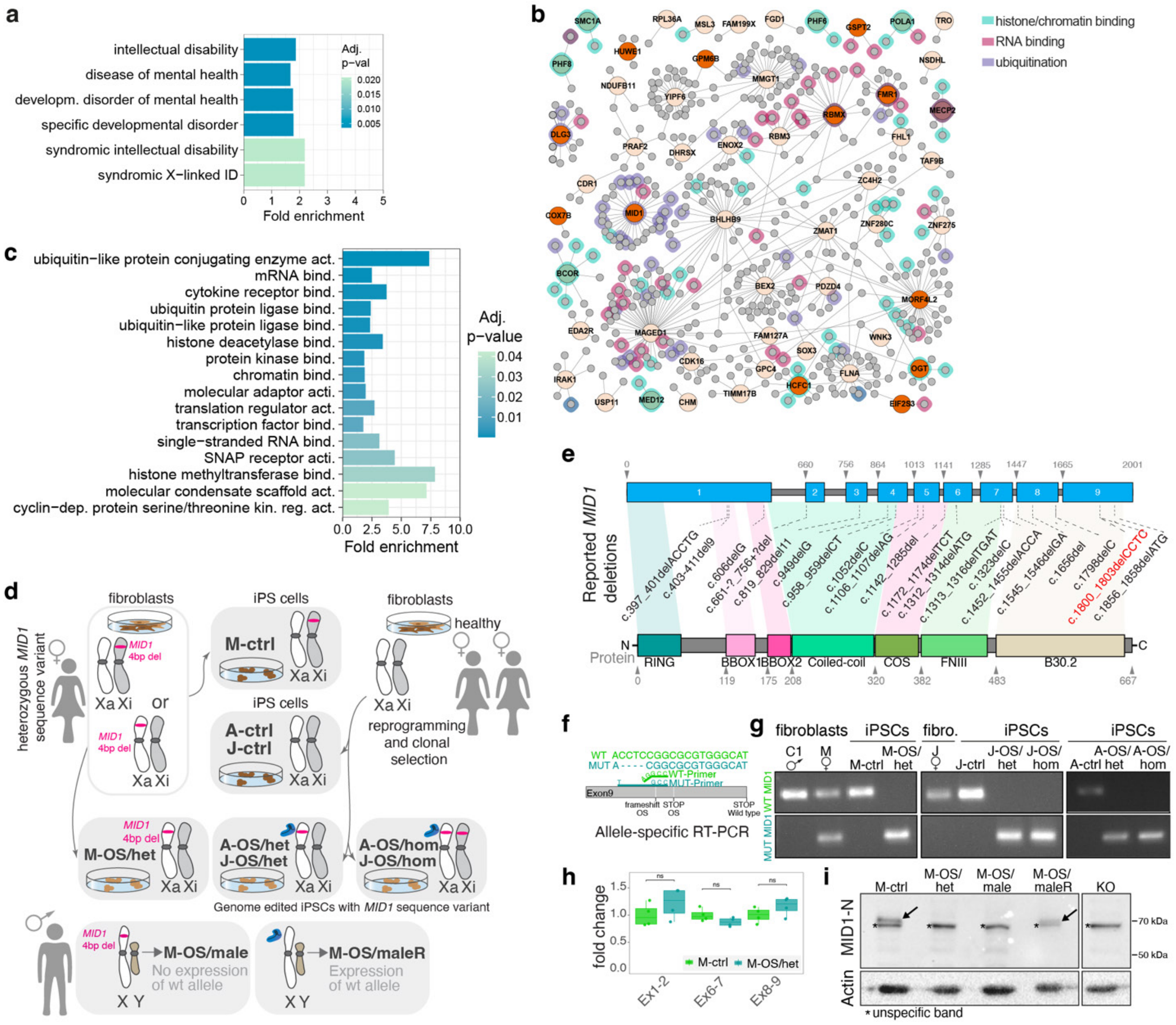

Fig. S5. Characterization of reactivated genes and dual allele usage in iPSC lines harboring heterozygous or homozygous *MID1* variants. a, Disease ontology enrichment analysis reveals an overrepresentation for X-linked intellectual disability and developmental disorders for reactivated genes. Statistical significance was tested using an upper-tail hypergeometric test with Benjamini-Hochberg corrections for multiple comparisons. **b,** A protein-protein interaction (PPI) network of protein-coding reactivated genes based on genes expressed in NPCs and neurons. Interactions between the PPI partners not involving reactivated genes are not shown. GO analysis revealed a significant overrepresentation of processes related to chromatin/histone binding, RNA binding and protein ubiquitination processes. Reactivated genes and their interaction partners that are involved in these processes are indicated on the network. Genes detected as reactivated in at

least one of the cell lines from the bulk RNA-seq analysis (A-ctr, J-ctrl or M-ctrl) were included in the analysis in a-c. **c,** Significantly overrepresented GO terms in the protein-protein interaction network of reactivated genes shown in Fig. S5b. An upper-tail hypergeometric test with Benjamini Hochberg correction for multiple comparisons was used to determine significance. **d,** Scheme depicting the logic of iPSC clone selection, carrying heterozygous 4-bp deletions in the X-chromosomal *MID1* gene, producing distinct iPSC lines from the same female donors with either one or the other X-chromosome active. Graphical scheme depicting the CRISPR/Cas9-mediated genome-editing of two wildtype female iPSC lines (J-ctrl, A-ctrl) in which the 4-bp deletion sequence variant was introduced hetero- or homozygously into the *MID1* gene (J-OS/het and J-OS/hom as well as A-OS/het and A-OS/hom, respectively). M-OS/het iPSCs were selected for expression of the 4-bp deletion variant on the active X-chromosome. The lowest panel shows the graphical depiction of the hemizygous 4-bp mutation in *MID1* in the M-OS/male iPSC line and the corresponding M/OS-maleR line in which the mutation was repaired. **e,** Scheme indicating MID1 gene and protein and highlighting currently known mutations in the *MID1* gene including the 4-bp deletion c.1800_1803delCCTC (in red) studied throughout this work. **f,** Primer sequences used for allele-specific RT-PCR. **g,** Allele-specific RT-PCR analysis of *MID1*-activation status in fibroblasts and M-iPSC lines (M-ctrl, M-OS/het), J-iPSC lines (J-ctrl, J-OS/het, J-OS/hom), and A-iPSC lines (A-ctrl, A-OS/het, A-OS/hom) derived from female fibroblasts (M, J, A). The C1 male control iPSC line shows the specificity of the mutant RT-PCR. **h,** qPCR was used to determine in iPSCs of the M-lines the mRNA stability of the *MID1* transcript in different exons. Fold-changes were calculated over the mean of M-ctrl (M-ctrl, n=4; M-OS/het, n=4; exact P values (left to right): 0.2, 0.2, 0.34). Statistical significance was calculated via two-sided Wilcoxon rank sum test. Dots represent samples from independent cultures. **i,** Protein levels of MID1 in M-iPSC lines as determined by western blot using an N-terminal MID1 antibody. An iPSC line with a CRISPR/Cas9-mediated knockout of the complete *MID1* gene reveals that the band indicated by a star is unspecific.

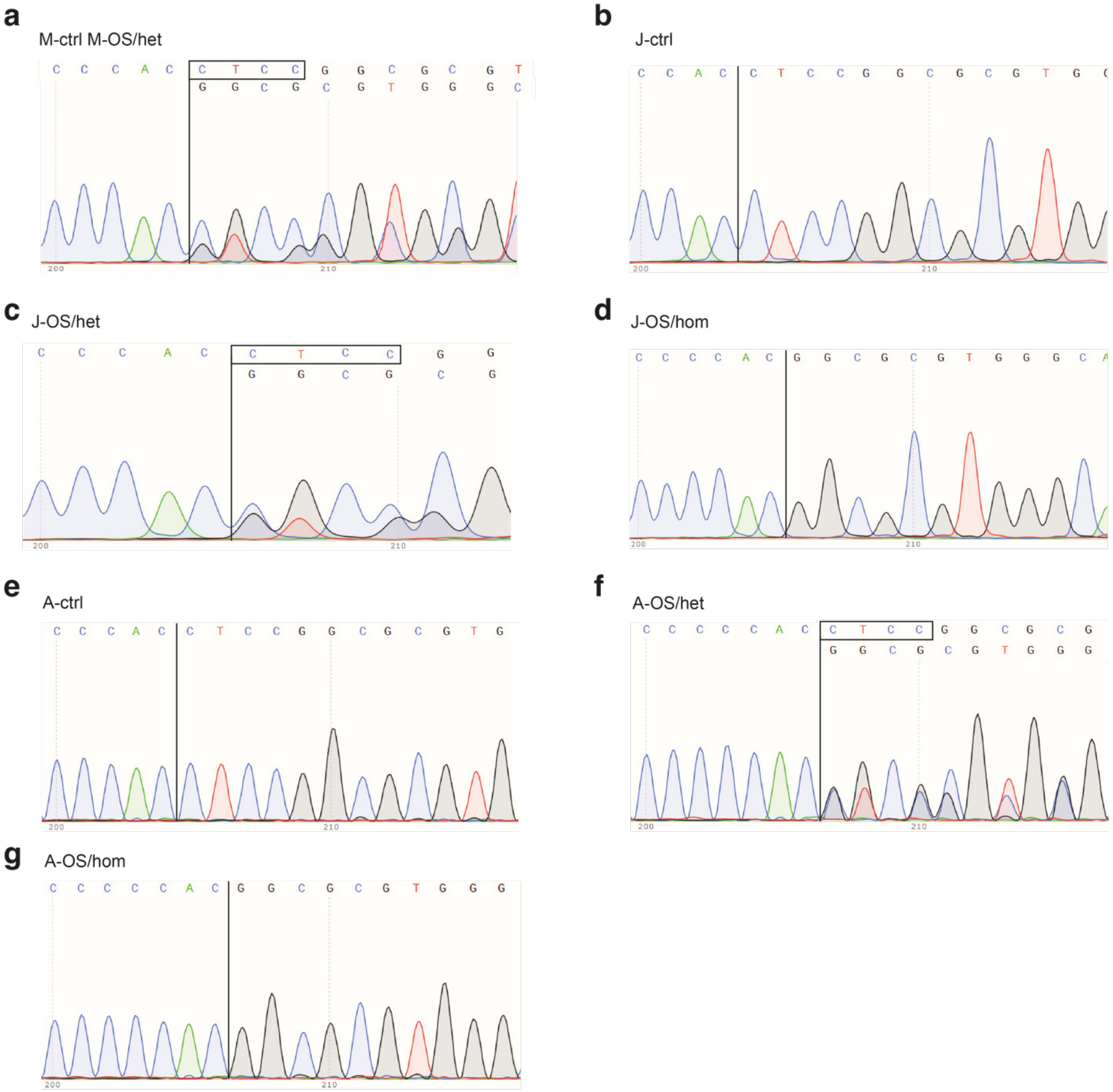

Fig. S6. Sanger sequencing chromatograms of the *MID1* gene in patient-derived and engineered iPSCs. a, Chromatogram from patient fibroblasts showing the frameshift mutation. The black line indicates its position, and the black box highlights the 4-bp deletion (c.1800_1803delCCTC). **b-d**, Wild-type J-ctrl iPSCs (**b**), heterozygous edited J-OS/het iPSCs (**c**), and homozygous edited J-OS/homo iPSCs (**d**). The black line marks the CRISPR gRNA cutting site; in panel **c**, the black box highlights the introduced 4-bp deletion. **e-g,** Wild-type A-ctrl iPSCs (**e**), heterozygous edited A-OS/het iPSCs (**f**), and homozygous edited A-OS/homo iPSCs (**g**),

shown analogously. The black line marks the gRNA cutting site, and panel **f** shows the introduced patient-specific deletion (c.1800_1803delCCTC) in the black box.

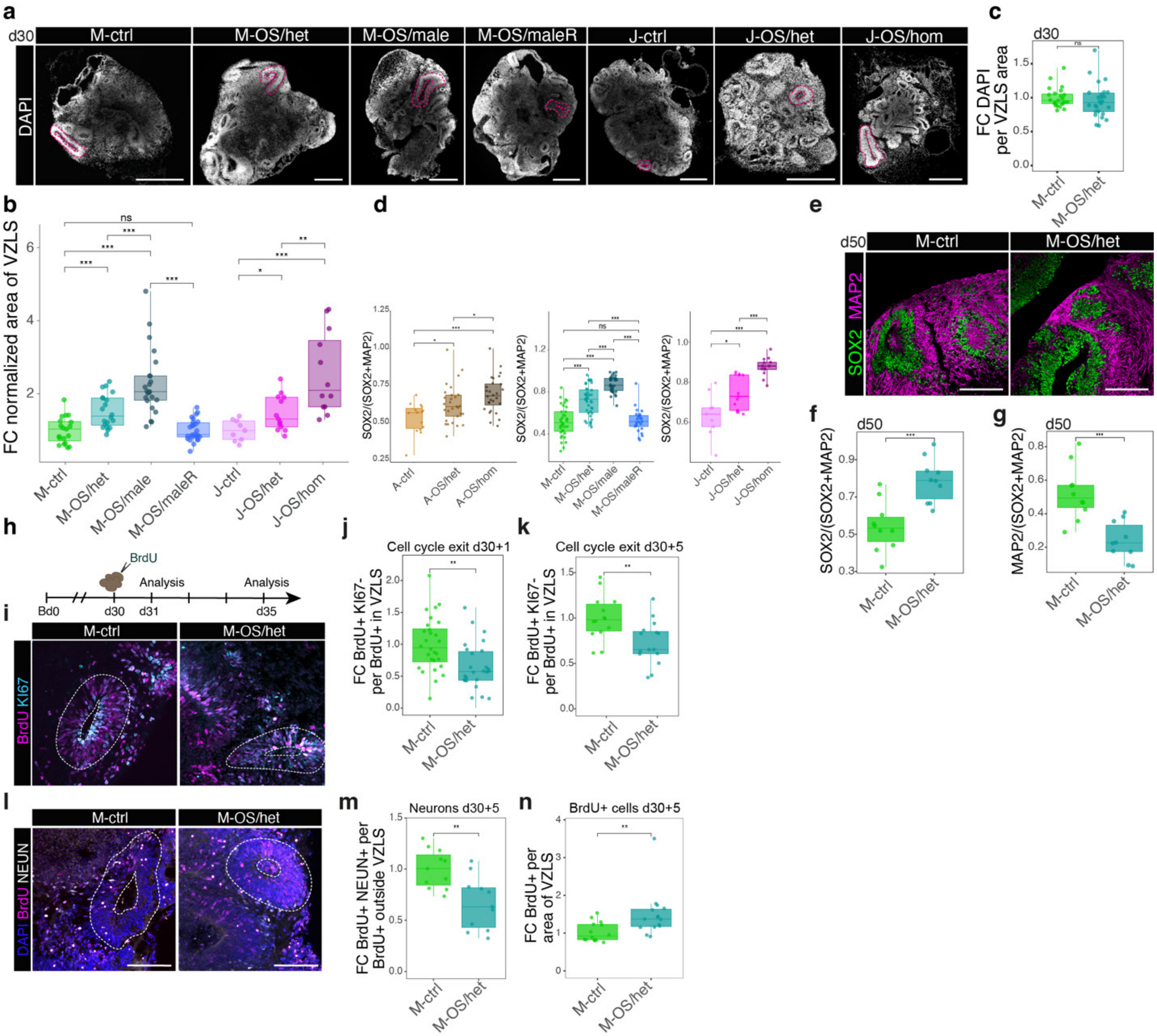

Fig. S7. Characterization of OS brain organoids and reactivation of *MID1* upon neural differentiation. a, Micrographs depicting d30 organoid sections stained with DAPI. The pink dashed lines highlight examples of VZLS quantified in Fig. S7b. Scale bar = 100 µm. **b,** Box- and jitter plot depicting the fold change of brain organoid area organized as VZLS compared to the respective ctrl line (M-ctrl, n=19; M-OS/het, n=20; M-OS/male, n=23; M-OS/maleR, n=26; J-ctrl, n=9; J-OS/het, n=13; J-OS/hom, n=12; exact P values (top to bottom): 0.83, $9.7*10^{-4}$, $4.5*10^{-3}$, $2.0*10^{-8}$, $2.7*10^{-5}$, $7.0*10^{-4}$, $2.5*10^{-2}$, $2.0*10^{-10}$). **c,** Quantification of the number of DAPI nuclei

per area within VZLS of brain organoids revealed no difference in cell density. Fold-change was calculated by dividing the cell density of each organoid by the mean cell density in controls (M-ctrl, n=23; M-OS/het, n=26; P=0.26). **d,** Box- and jitter plot depicting the quantification of the fraction of SOX2+ area per total neural area (SOX2+ or MAP2+ area) in d30 organoids. Left: (A-ctrl, n=15; A-OS/het, n=29; A-OS/hom, n=28; exact P values (top to bottom): 0.035, $8*10^{-5}$, 0.032). Middle: (M-ctrl, n=36; M-OS/het, n=39; M-OS/male, n=34; M-OS/maleR, n=25; exact P values (top to bottom): $7.5*10^{-7}$, 0.71, $1.4*10^{-13}$, $2.8*10^{-7}$, $<2.2*10^{-16}$, $1.3*10^{-7}$). Right: (J-ctrl, n=9; J-OS/het, n=13; J-OS/hom, n=13; exact P values (top to bottom): $3.7*10^{-5}$, $8.0*10^{-6}$, $2.1*10^{-2}$). **e,** Micrographs showing d50 brain organoid sections stained for SOX2 (green) and MAP2 (magenta). Scale bar=100 µm. **f,** Quantification of the fraction of SOX2 in neural tissue (SOX2+MAP2+ area) in d50 organoids (M-ctrl, n=10; M-OS/het, n=10; P=$3.2*10^{-4}$). **g,** Quantification of the fraction of MAP2 in neural tissue (SOX2+MAP2+ area) in d50 organoids (M-ctrl, n=10; M-OS/het, n=10; P=$3.2*10^{-4}$). **h,** Experimental scheme depicting the BrdU labeling experiment used to assess cell cycle exit of NPCs in d30 brain organoids. **i,** Micrographs showing representative stainings for BrdU (magenta) and KI67 (light blue) within VZLS (highlighted with white dashed lines) 24 hours following BrdU pulse. Scale bar=100µm. **j,** Boxplot showing the quantification of the number of $BrdU^+KI67^-$ cells per total number of $BrdU^+$ cells quantified within VZLS 24 hours after BrdU addition. (M-ctrl, n=28; M-OS/het, n=26; P=$1.7*10^{-3}$). **k,** Boxplot showing the quantification of the number of $BrdU^+KI67^-$ cells per total number of $BrdU^+$ cells quantified within VZLS 5 days after BrdU addition. (M-ctrl, n=14; M-OS/het, n=15; P=$7.9*10^{-3}$) **l**, Micrographs of brain organoid sections stained for DAPI (blue), BrdU (magenta), and NEUN (white) 5 days following BrdU addition. VZLS are highlighted with white dashed lines. Scale bar=100 µm. **m**, Boxplot showing the quantification of $BrdU^+$ $NEUN^+$ cells per total $BrdU^+$ cells quantified outside of VZLS 5 days following BrdU addition. (M-ctrl, n=11; M-OS/het, n=11; P=$5.2*10^{-3}$). **n,** Boxplot depicting the quantification of $BrdU^+$ cells per area of VZLS 5 days following BrdU addition. (M-ctrl, n=14; M-OS/het, n=15; P=$3.2*10^{-3}$). For **b, c, j, k, m, n,** Fold-change was calculated by dividing the value for each organoid by the average value of controls in the respective batch. For **b, c, d, f, g, j, k, m, n** boxplots show median, quartiles (box), and range (whiskers). In the jitter plots dots represent individual organoids. Two-sided Wilcoxon rank sum test was used; *P<0.05, **P<0.01, ***P<0.001, ns P>0.05.

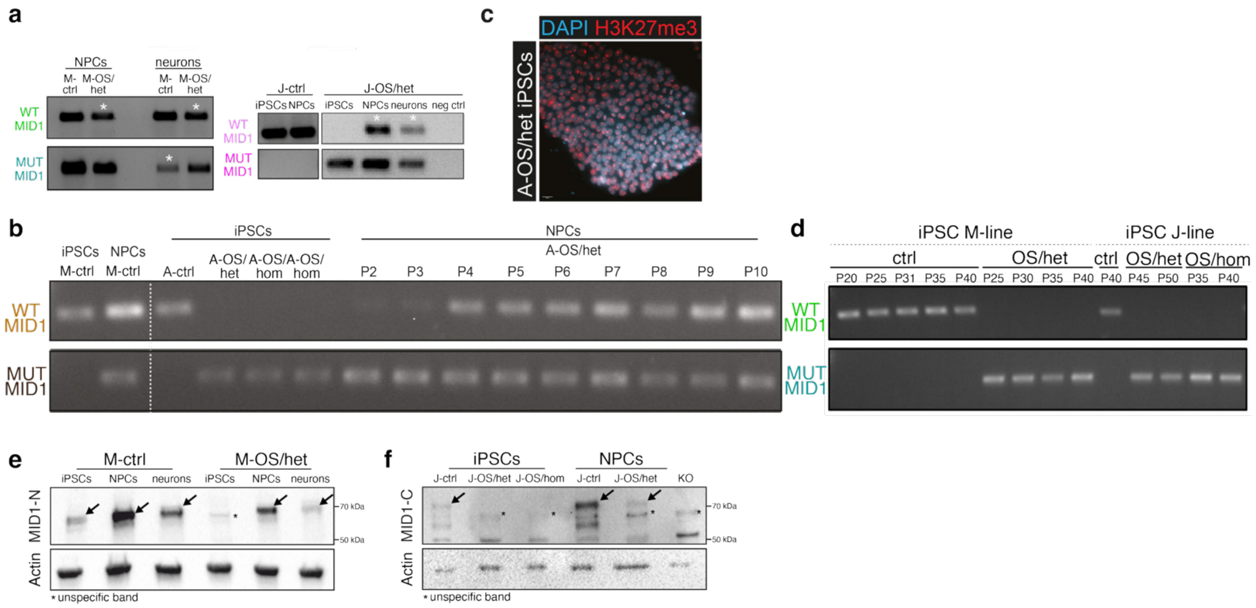

Fig. S8. Reactivation of MID1 during neural differentiation. a, Allele-specific RT-PCR showing the expression of the mutant and wildtype allele in NPCs and neurons derived from M-ctrl and M-OS/het iPSC lines as well as in iPSCs and NPCs of J-ctrl and J-OS/het. **b,** Allele-specific RT-PCR confirming reactivation of the second *MID1* allele in M-ctrl as well as in A-OS/het NPCs with increasing passages. **c,** Immunofluorescence stainings using an antibody against H3K27me3 (red) in DAPI positive nuclei (blue) of A-OS/het line iPSCs. Scale bars =20µm. **d,** Allele-specific RT-PCR showing the expression of the mutant and wildtype allele in NPCs and neurons derived from M-ctrl and M-OS/het iPSC lines as well as in iPSCs and NPCs of J-ctrl and J-OS/het across increasing passages. **e,** Protein levels of MID1 in M-ctrl and M-OS/het iPSCs, NPCs and neurons as determined by western blot using an N-terminal MID1 antibody. **f**, Western blot showing protein levels of MID1 in J-ctrl, J-OS/het, and J-OS/hom iPSCs and NPCs using a C-terminal MID1 antibody. An iPSC line with a CRISPR/Cas9-mediated knockout of the complete *MID1* gene reveals that the band indicated by a star is unspecific. **e, f,** actin was used as a loading control.

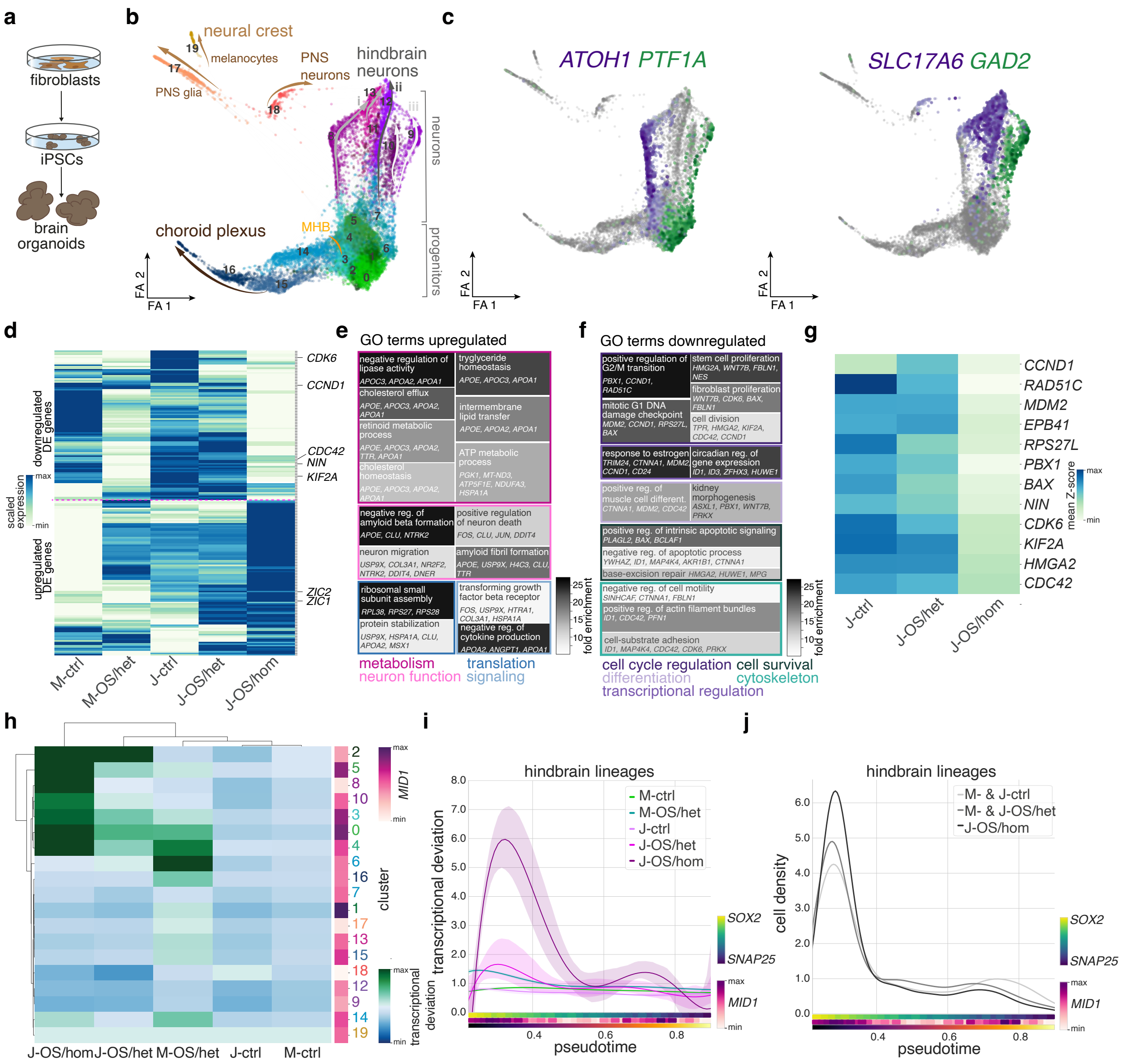

Fig. S9. Transcriptional characterization of brain organoids derived from patient and genome edited iPSC lines with *MID1* mutations. a, Experimental scheme. **b,** Force-directed graph embedding and Leiden clusters (indicated with numbers) of the neural cells (excluding clusters 1, 3, 11, 14, 16, 20 from the entire data set as shown in Fig. 5c) highlighting various differentiation trajectories. Note the main three distinct neuronal hindbrain lineages i) glutamatergic neurons, ii) noradrenergic neurons, and iii) GABAergic neurons. Number of cells total: 11923, M-ctrl: 2017; M-OS/het: 4366; J-ctrl: 1565; J-OS/het: 615; J-OS/hom: 3360. **c,** Lineage reconstruction showing the mutually exclusive trajectories of rhombic lip progenitors (*ATOH1*) to glutamatergic cells (*SLC17A6*) and ventricular zone progenitors (*PTF1A*) to GABAergic cells (*GAD2*). In **b, c,** FA refers to force atlas. **d,** Heatmap showing genes

differentially expressed in NPCs (clusters 1, 2, 3, 4, 5, 6 in Fig. 6a) between J-ctrl and J-OS/hom. Note an intermediate change in expression in J-OS/het compared to J-ctrl and J-OS/hom. **e, f**, Top 15 GO terms enriched in the differentially expressed genes in NPCs. GO terms were grouped as indicated below the tileplot. The color of the individual tiles indicates the fold enrichment. **g,** Heatmap showing expression of selected cell cycle related genes in ctrl and OS conditions. Note the intermediate expression pattern in J-OS/het versus J-OS/hom. **h,** Heatmap showing the transcriptional deviation in each cluster from the respective control line and the corresponding *MID1* expression level. The dendrogram clustering the samples separates them according to their genotype with heterozygous lines showing intermediate deviation. There is no apparent correlation between the level of *MID1* expression and the degree of transcriptional deviation. **i,** Fitted line plot showing the pseudotemporal ordering of the transcriptional deviation in each cell along the hindbrain differentiation trajectory in different genotypes with the 95% confidence interval as a transparent band of the same color in the background. For orientation purposes the plot includes *MID1* expression as well as the *SOX2*/*SNAP25* ratio (NPCs versus neurons) averaged in 50 equally sized pseudotemporal bins. Besides the intermediate transcriptional deviation in heterozygous lines this plot reveals that the divergence in both heterozygous and homozygous lines peaks at NPC stages. **j,** Density distribution plot highlighting the accumulation of mutant cells at NPC stages.

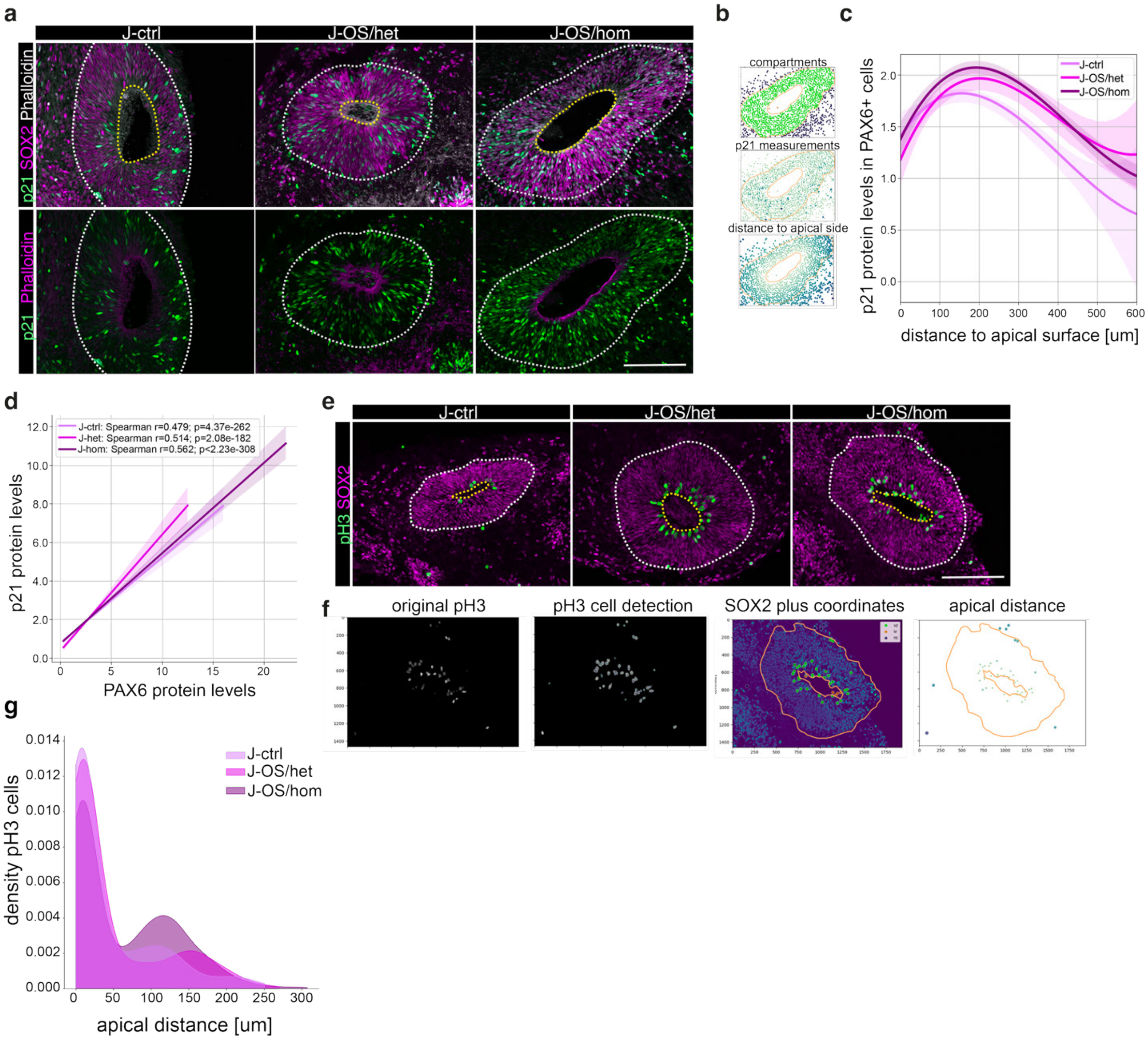

Fig. S10. Molecular mechanism underlying gradual progenitor phenotype. a, Representative images of J-ctrl, J-OS/het, and J-OS/hom derived brain organoid slices stained with antibodies against p21, SOX2 and Phalloidin. The basal and the apical side of VZLS are indicated by white and yellow dashed lines, respectively. Scale bar = 100 µm. **b,** Result of image processing and nuclei segmentation including outline of the VZLS. This allows quantification of p21 protein levels in PAX6 positive cell nuclei in relation to the distance to the apical surface. **c,** Fitted line plot depicting the p21 protein levels quantified in VZLS resident PAX6 positive cells in relation to the apical side. 95% confidence interval is shown as a transparent band in the same respective color. **d,** Linear correlation plot of PAX6 and p21 protein levels in individual cells across experimental conditions. 95% confidence interval is shown as a transparent band in the same

respective color. For **c, d,** *n*=5 organoids for J-ctrl, *n*=6 organoids for J-OS/het, J/OS-hom. J-ctrl=4705 cells, J/OS-het=2701 cells, J/OS-hom=6602 cells. **e,** Images of VZLS within brain organoids derived from J-ctrl, J-OS/het and J-OS/hom iPSC lines stained for pH3 and SOX2. SOX2 was used to label progenitors within VZLS and pH3 was used to visualize dividing cells, the location of which was quantified by a python-based image analysis pipeline shown in b. The basal and the apical side of VZLS are indicated by white and yellow dashed lines, respectively. Scale bar = 100 µm. **f,** Example of python-based image analysis allowing segmentation and allocation of pH3 positive cells within the VZLS. **g,** Density distribution of pH3 positive cells along apical distance within VZLS across experimental conditions. *n*=6 organoids in all conditions. J-ctrl=153 cells, J/OS-het=210 cells, J/OS-hom=285 cells.

Source Data
Uncropped scans of blots and gels within Supplementary Figures

Supplementary Fig. S5g
MID1 allele-specific RT-PCR (left panel)
Loading:

1*	2*	3*	4*	5	6*	7*	8*	9*	10	11
C1 fibroblasts	M fibroblasts	M-ctrl iPSCs	M-OS/het iPSCs	negative ctrl	J fibroblasts	J-ctrl iPSCs	J-OS/het iPSCs	J-OS/hom iPSCs	negative ctrl	empty

Samples indicated with * are shown inside manuscript Figures.
PCRs of samples in the upper part of the gel were run with MID1 WT primers.
PCRs of samples in the upper part of the gel were run with MID1 MUT primers.

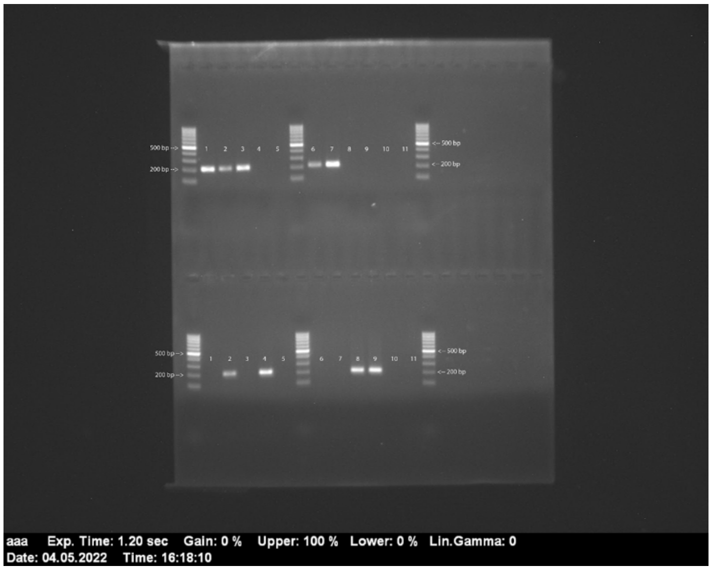

MID1 allele-specific RT-PCR (right panel)

Loading:

1	2	3*	4*	5*	6
M-ctrl iPSCs	M-OS/het iPSCs	A-ctrl iPSCs	A-OS/het iPSCs	A-OS/hom iPSCs	A-OS/hom iPSCs

Samples indicated with * are shown inside manuscript Figures.

PCRs of samples in the upper part of the gel were run with MID1 WT primers.

PCRs of samples in the upper part of the gel were run with MID1 MUT primers.

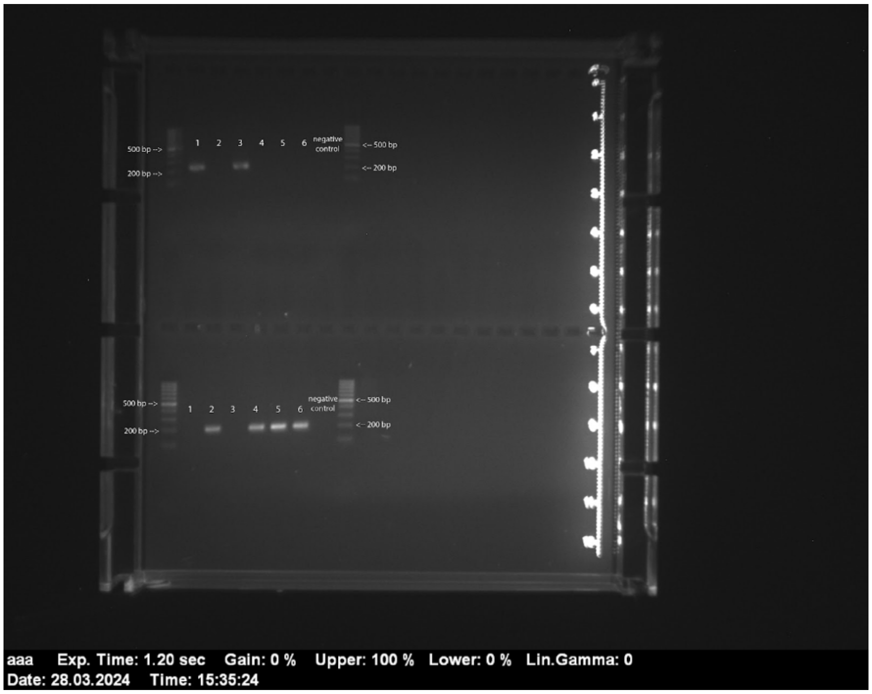

Supplementary Fig. S5i
MID1 Western Blot
Loading:

1*	2*	3*	4*	5	6
M-ctrl iPSCs	M-OS/het iPSCs	M-OS/male iPSCs	M-OS/maleR iPSCs	M-ctrl compound het. iPSCs	C1 KO

Samples indicated with * are shown inside manuscript Figures.
Bands of samples in the upper blot were incubated with the antibody against the N-terminal MID1.
Bands of samples in the lower blot were incubated with the antibody against beta-actin.

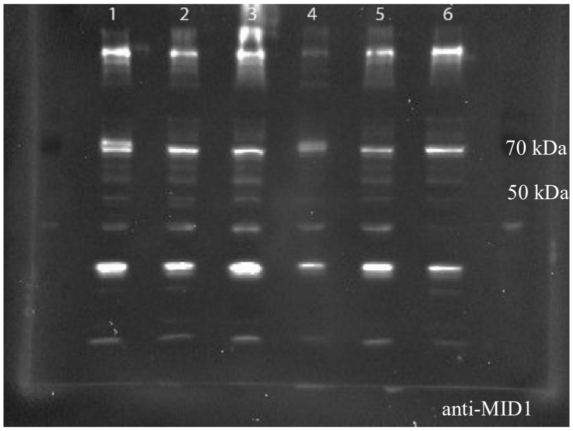

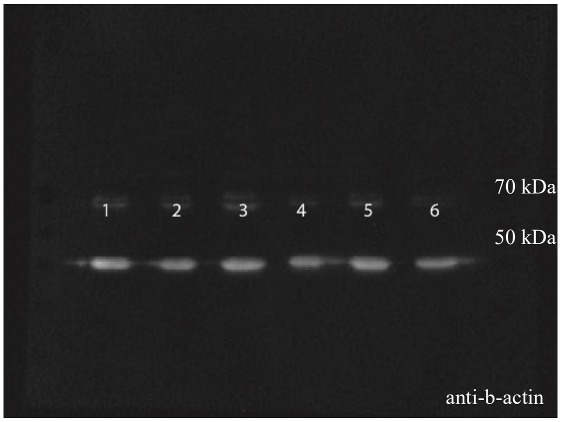

Supplementary Fig. S8a
MID1 allele-specific RT-PCR (left panel)
Loading:

1	2	3	4	5	6	7	8	9*	10*	11	12*	13*
negative ctrl	C1 fibro	M-OS male fibro	M-line fibroblasts	empty	M-ctrl iPSCs	M-OS/het iPSCs	empty	M-ctrl NPCs	M-OS het NPCs	empty	M-ctrl neurons	M-OS /het neurons

Samples indicated with * are shown inside manuscript Figures.
PCRs of samples in the upper part of the gel were run with MID1 WT primers.
PCRs of samples in the upper part of the gel were run with MID1 MUT primers.

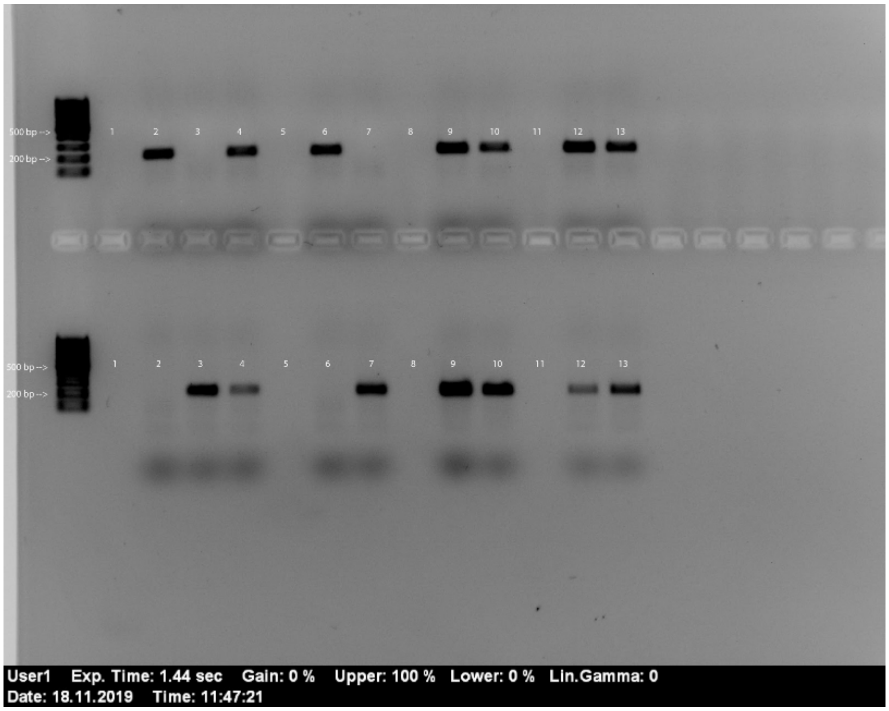

MID1 allele-specific RT-PCR (middle panel)
Loading:

1*	2*	3	4	5	6	7	8	9	10	11	12	13	14	15
J-ctrl iPSC s	J-ctrl NPC s	J-OS het iPSC s	J-OS het NPCs passag e 4	J-OS het NPCs passag e 6	J-OS het NPCs passag e 7	M-ctrl organ oids	M-ctrl organ oids	M-ctrl organoi ds	M-ctrl organoi ds	M-ctrl organoids d30 dissociate d cells	M-ctrl organoids d60 dissociate d cells	M-ctrl NPCs from organoi ds	M-ctrl neurons from organodi s	

Samples indicated with * are shown inside manuscript Figures.
PCRs of samples in the upper part of the gel were run with MID1 WT primers.
PCRs of samples in the upper part of the gel were run with MID1 MUT primers.

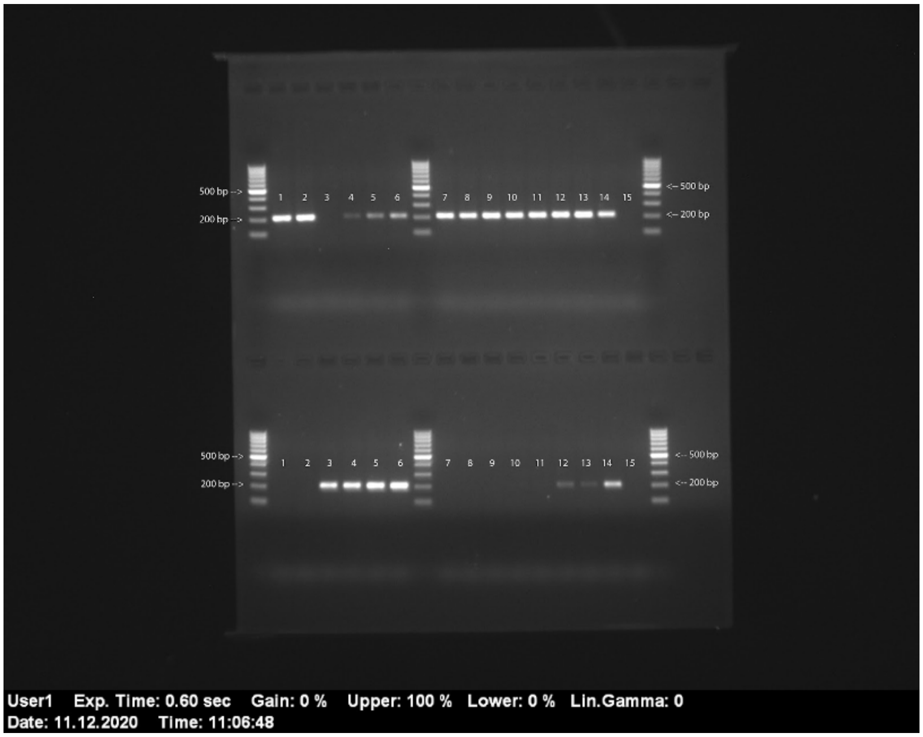

MID1 allele-specific RT-PCR (right panel)

Loading:

1	2	3	4	5	6	7*	8*	9*	10*
M fibroblasts	M-ctrl iPSCs	M-ctrl NPCs	J-ctrl iPSCs	J-OS/hom iPSCs	J-ctrl NPCs	J-OS/het iPSCs	J-OS/het NPCs	J-OS/het neurons	negative ctrl

Samples indicated with * are shown inside manuscript Figures.

PCRs of samples in the upper part of the gel were run with MID1 WT primers.

PCRs of samples in the upper part of the gel were run with MID1 MUT primers.

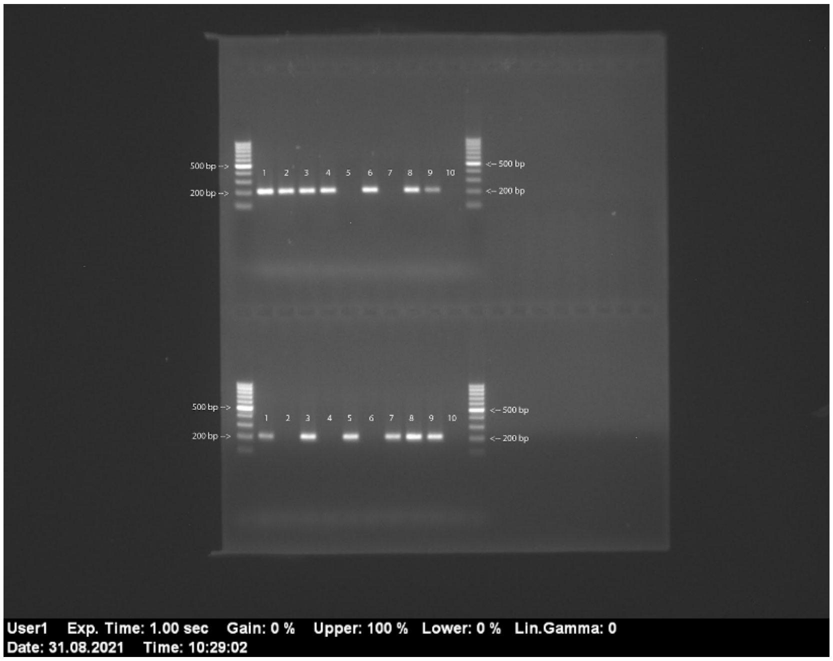

Supplementary Fig. S8b
MID1 allele-specific RT-PCR
Loading:

1*	2*	3*	4*	5*	6*	7*	8*	9*	10*	11*	12*	13*	14*	15*
M-ctrl iPSCs	M-ctrl NPCs	A-ctrl iPSCs	A-OS/het iPSCs	A-OS/hom iPSCs	A-OS/hom iPSCs	A-OS/het NPCs p2	A-OS/het NPCs p3	A-OS/het NPCs p4	A-OS/het NPCs p5	A-OS/het NPCs p6	A-OS/het NPCs p7	A-OS/het NPCs p8	A-OS/het NPCs p9	A-OS/het NPCs p10

Samples indicated with * are shown inside manuscript Figures.
PCRs of samples in the upper part of the gel were run with MID1 WT primers.
PCRs of samples in the upper part of the gel were run with MID1 MUT primers.

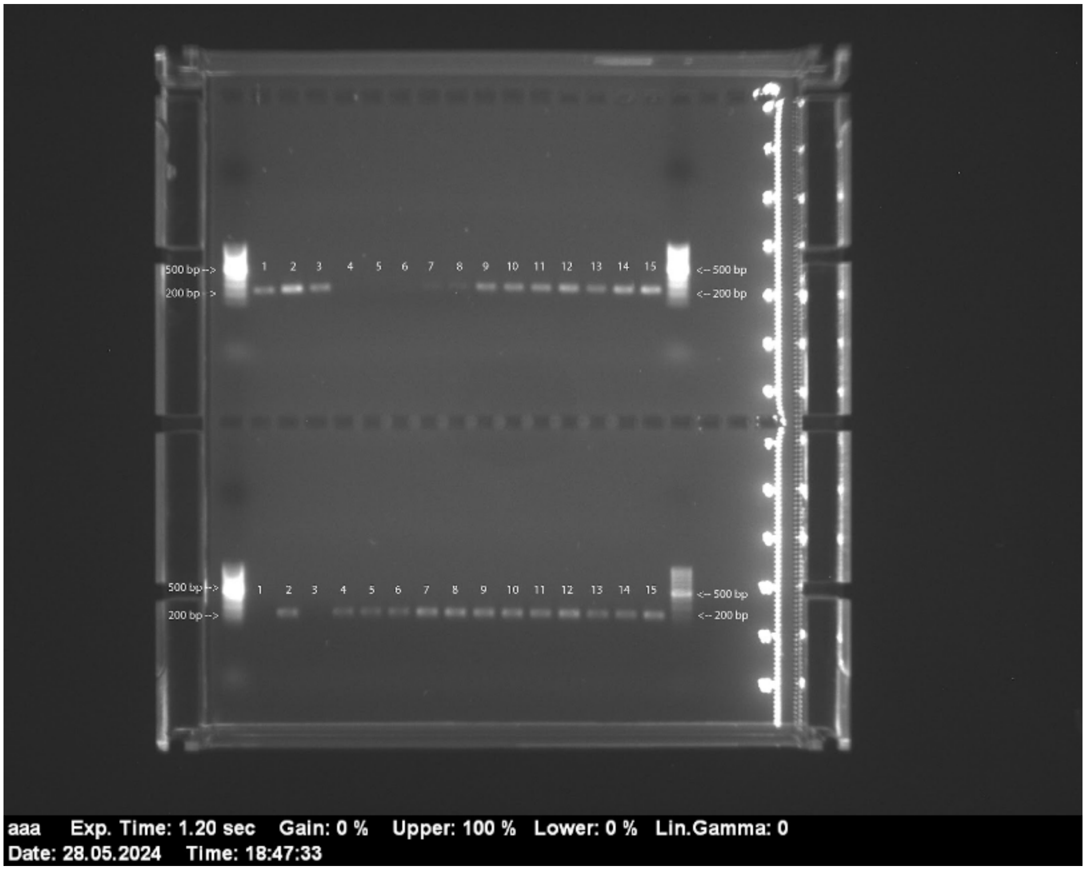

Supplementary Fig. S8d

MID1 allele-specific RT-PCR

Loading:

1*	2*	3*	4*	5*	6*	7*	8*	9*	10*	11*	12*	13*	14*	15	16	17	18	19
M-ctrl iPSCs p20	M-ctrl iPSCs p25	M-ctrl iPSCs p31	M-ctrl iPSCs p35	M-ctrl iPSCs p40	M-OS/het iPSCs p25	M-OS/het iPSCs p30	M-OS/het iPSCs p35	M-OS/het iPSCs p40	J-ctrl iPSCs p40	J-OS/het iPSCs p45	J-OS/het iPSCs p50	J-OS/hom iPSCs p35	J-OS/hom iPSCs p40	M fibroblasts	M-ctrl iPSCs	M-ctrl NPCs	M-ctrl neurons	neg ctrl

Samples indicated with * are shown inside manuscript Figures.

PCRs of samples in the upper part of the gel were run with MID1 WT primers.

PCRs of samples in the upper part of the gel were run with MID1 MUT primers.

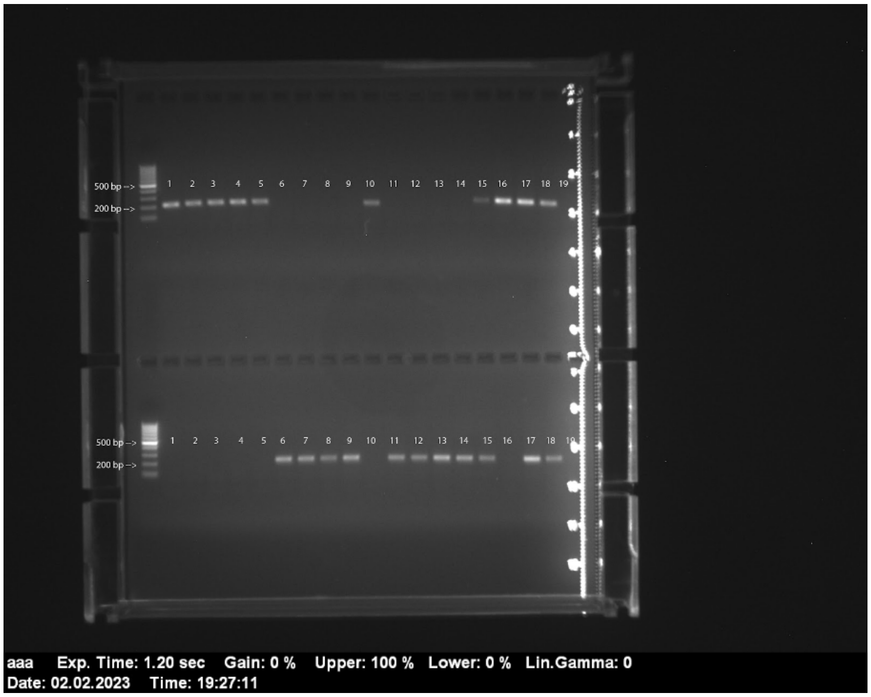

Supplementary Fig. S8e

MID1 Western Blot

Loading:

1*	2*	3*	4*	5*	6*
M-ctrl iPSCs	M-ctrl NPCs	M-ctrl neurons	M-OS het iPSCs	M-OS het NPCs	M-OS het neurons

Samples indicated with * are shown inside manuscript Figures.

Bands of samples in the upper blot were incubated with the antibody against the N-terminal MID1.

Bands of samples in the lower blot were incubated with the antibody against beta-actin.

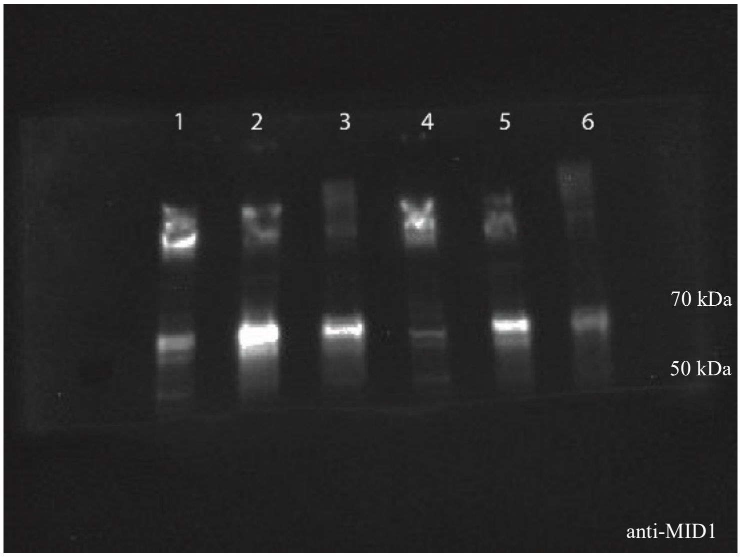

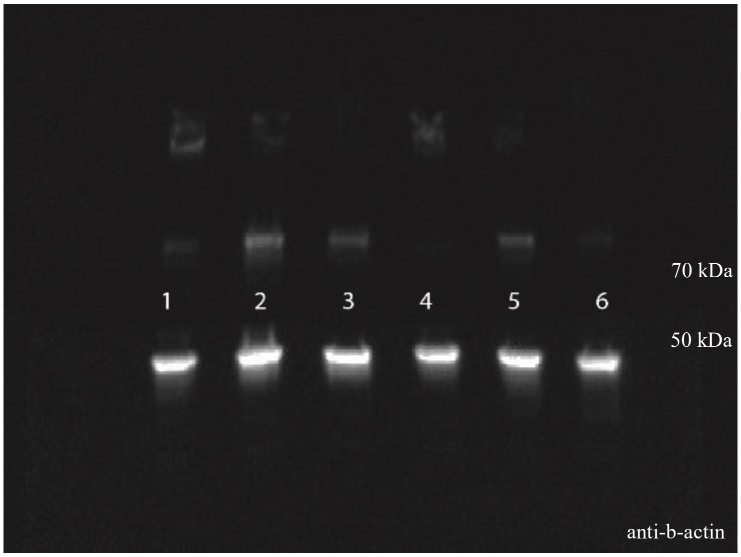

Supplementary Fig. S8f
MID1 Western Blot
Loading:

1*	2*	3*	4*	5*	6*
J-ctrl iPSCs	J-OS het iPSCs	J-OS homo iPSCs	J-ctrl NPCs	J-OS het NPCs	C1 KO

Samples indicated with * are shown inside manuscript Figures.
Bands of samples in the upper blot were incubated with the antibody against the C-terminal MID1.
Bands of samples in the lower blot were incubated with the antibody against beta-actin.

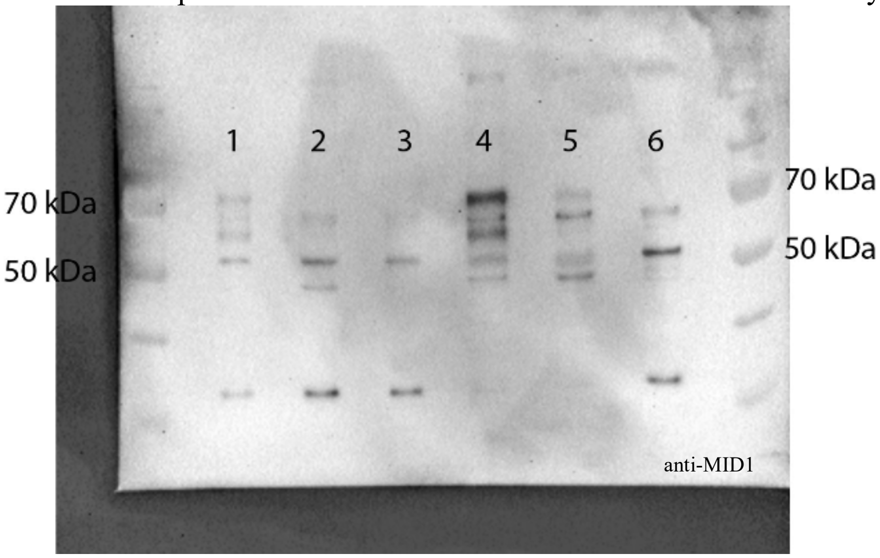

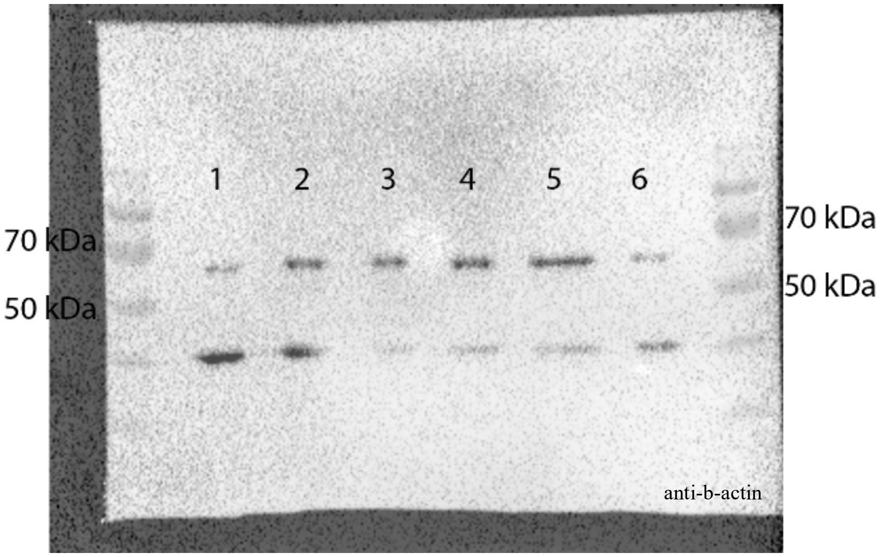

Supplement: Supplementary file 1 — Supplementary Information [file 41467_2026_68428_MOESM1_ESM.pdf]
