# Supplementary material for: Dynamic allele usage of X-linked genes ameliorates neurodevelopmental disease phenotypes in brain organoids
Source: Nat Commun. 2026 Jan 14;17:599. doi: 10.1038/s41467-026-68428-x (PMC12808108; doi:10.1038/s41467-026-68428-x)
Supplement: Supplementary file 2 — Description of Additional Supplementary Files [file 41467_2026_68428_MOESM2_ESM.pdf]

Description of Additional Supplementary Files

File name: Supplementary Data 1
Description: Overview of iPSC lines used in this study.

File name: Supplementary Data 2
Description: Comparison of biallelically expressed genes to other previously published data sets.

File name: Supplementary Data 3
Description: GO terms in the PPI network of reactivated genes shown in Fig. S5.

File name: Supplementary Data 4
Description: List of genes included in the heatmap shown in Fig. 4i.

File name: Supplementary Data 5
Description: Sequences of oligonucleotides used in this study.
