# Supplementary material for: Dynamic allele usage of X-linked genes ameliorates neurodevelopmental disease phenotypes in brain organoids
Source: Nat Commun. 2026 Jan 14;17:599. doi: 10.1038/s41467-026-68428-x (PMC12808108; doi:10.1038/s41467-026-68428-x)
Supplement: Supplementary file 8 — Reporting Summary [file 41467_2026_68428_MOESM8_ESM.pdf]

nature portfolio

Corresponding author(s): Schweiger, Falk, Karow

Last updated by author(s): Dec 16, 2025

Reporting Summary

Nature Portfolio wishes to improve the reproducibility of the work that we publish. This form provides structure for consistency and transparency in reporting. For further information on Nature Portfolio policies, see our Editorial Policies and the Editorial Policy Checklist.

Statistics

For all statistical analyses, confirm that the following items are present in the figure legend, table legend, main text, or Methods section.

n/a	Confirmed	
☐	☒	The exact sample size (*n*) for each experimental group/condition, given as a discrete number and unit of measurement
☐	☒	A statement on whether measurements were taken from distinct samples or whether the same sample was measured repeatedly
☐	☒	The statistical test(s) used AND whether they are one- or two-sided<br>*Only common tests should be described solely by name; describe more complex techniques in the Methods section.*
☐	☒	A description of all covariates tested
☐	☒	A description of any assumptions or corrections, such as tests of normality and adjustment for multiple comparisons
☐	☒	A full description of the statistical parameters including central tendency (e.g. means) or other basic estimates (e.g. regression coefficient) AND variation (e.g. standard deviation) or associated estimates of uncertainty (e.g. confidence intervals)
☐	☒	For null hypothesis testing, the test statistic (e.g. *F*, *t*, *r*) with confidence intervals, effect sizes, degrees of freedom and *P* value noted<br>*Give P values as exact values whenever suitable.*
☐	☒	For Bayesian analysis, information on the choice of priors and Markov chain Monte Carlo settings
☒	☐	For hierarchical and complex designs, identification of the appropriate level for tests and full reporting of outcomes
☒	☐	Estimates of effect sizes (e.g. Cohen's *d*, Pearson's *r*), indicating how they were calculated

Our web collection on statistics for biologists contains articles on many of the points above.

Software and code

Policy information about availability of computer code

Data collection	Details about data collection including a description of the R and python packages used in this study are included in the methods section of the manuscript and provided here. PyroMark Assay Design Software (v2.0), VisiView software (v7.0.0.7)
Data analysis	All computational tools (code) used in this study are publicly available. The following computational packages were employed: Imaris Software (v10), FIJI (v1.52-1.53), R (v3.5.1-4.1.2), OpenCV (v4.4.0 - 4.5.1), python (v3.9.1-3.9.10), NumPy (v1.21.4, v1.21.5), pandas (v1.3.4, v 3.9.7), CellPose (v2.2.3),scikit-image package (v0.21.0), python (v3.8.19), celldetection (v0.4.9), bcl2fastq (v2.17.1.14) Cutadapt (v0.18), FastQC (v0.11.7), STAR aligner (v2.5.3, v2.7.8a), nudup.py (v2.3), BBDuk tool from BBMap (v38.86), FastQC (v0.11.9), STAR v2.7.10b, Subread (v2.0.1),<br>NVIDIA Clara Parabricks (v3.5), stats (v4.0.2), meta (v4.18.1), karyoploteR (v1.4.1), DESeq2 (v1.42.1), igraph (v1.2.6), clusterProfiler (v3.16.1), ChromDiff tool, deepTools (v3.5.4), DOSE (v3.28.2), Cell Ranger (v4.0.0 – 6.0.2, v.7.1.0-8.0.0), Souporcell (v2.5), Scanpy (v1.8.1, v1.8.2), Scrublet (v0.2.3), SciPy (v1.7.0), bbknn’s (v1.4.0), fa2 (v3.5), Leiden (v0.8.7), samtools (v1.10), velocyto (v0.17), scVelo (v0.2.3), TopGO (v2.44.0), fasterq-dump (v3.0.0), FreeBayes (v1.3.5)

For manuscripts utilizing custom algorithms or software that are central to the research but not yet described in published literature, software must be made available to editors and reviewers. We strongly encourage code deposition in a community repository (e.g. GitHub). See the Nature Portfolio guidelines for submitting code & software for further information.

Data

Policy information about availability of data

All manuscripts must include a data availability statement. This statement should provide the following information, where applicable:

- Accession codes, unique identifiers, or web links for publicly available datasets
- A description of any restrictions on data availability
- For clinical datasets or third party data, please ensure that the statement adheres to our policy

The bulk RNAseq data is deposited in the Sequence Read Archive (SRA) under the BioProject PRJNA819272 [https://www.ncbi.nlm.nih.gov/bioproject/?term=PRJNA819272]. The FASTQ files of the single nucleus and single cellRNA-seq data are deposited in the European Nucleotide Archive (ENA) at EMBL-EBI under accession number PRJEB96835 [https://www.ebi.ac.uk/ena/browser/view/PRJEB96835]. Source data are provided as a Source Data file with this paper.

Research involving human participants, their data, or biological material

Policy information about studies with human participants or human data. See also policy information about sex, gender (identity/presentation), and sexual orientation and race, ethnicity and racism.

Reporting on sex and gender	In this manuscript we distinguish between the sex of cells and base this classification on the composition of gonosomes. We provide the information of the sex (composition of gonosomes) of all cells used in the study in Table S1. The findings in this study apply to XX females.
Reporting on race, ethnicity, or other socially relevant groupings	We did not group participants into social categories.
Population characteristics	The covariate-relevant population characteristics of the participants are summarized and provided in Table S1.
Recruitment	Participants were recruited in the Human Genetics Departments of the Universities in Bristol and Mainz and recruited based on the composition of their gonosomes.
Ethics oversight	Ethical approval<br>Patients' fibroblasts to generate M-ctrl, M-OS/het, M-OS/male, M-OS/maleR iPSCs were established in Bristol Genetics Laboratory, following consent for further analysis and usage for research in an anonymized way was given by the family. Fibroblasts to generate J-ctrl, J2-ctrl, J-OS/het, J-OS/hom, J2-ctrl, the A-ctrl, A-OS/het, A-OS/hom, C1-male, and the KO-line iPSCs were taken at the University Medical Center in Mainz following approval by the local ethical committee (No. 4485). Consent for further analysis and usage for research in an anonymized way was given.

Note that full information on the approval of the study protocol must also be provided in the manuscript.

Field-specific reporting

Please select the one below that is the best fit for your research. If you are not sure, read the appropriate sections before making your selection.

☒ Life sciences ☐ Behavioural & social sciences ☐ Ecological, evolutionary & environmental sciences

For a reference copy of the document with all sections, see nature.com/documents/nr-reporting-summary-flat.pdf

Life sciences study design

All studies must disclose on these points even when the disclosure is negative.

Sample size	No statistical methods were used to predetermine sample sizes. Sample sizes were predetermined on the basis of published studies (PMID: 38718796). Unless stated differently, quantitative experiments in this study were repeated at least three times.
Data exclusions	Criteria for exclusion of single cells from sc/snRNA-seq analysis are described in the methods section under heading "Single nucleus and single cell RNA-seq data preprocessing, clustering, visualization"
Replication	For all experiments all replicates are indicated in the figures and figure legends or the methods sections. All replications were successful.
Randomization	Not applicable to this manuscript. Groups were defined by genotype or differentiation state. The order of data collection was randomized.
Blinding	(sc)RNA-sequencing analyses were performed unbiasedly and therefore blinding is not applicable. Quantifications of organoid images were performed using an automated analysis pipeline therefore blinding was not relevant.

Reporting for specific materials, systems and methods

We require information from authors about some types of materials, experimental systems and methods used in many studies. Here, indicate whether each material, system or method listed is relevant to your study. If you are not sure if a list item applies to your research, read the appropriate section before selecting a response.

Materials & experimental systems

n/a	Involved in the study
☐	☒ Antibodies
☐	☒ Eukaryotic cell lines
☒	☐ Palaeontology and archaeology
☐	☒ Animals and other organisms
☒	☐ Clinical data
☒	☐ Dual use research of concern
☒	☐ Plants

Methods

n/a	Involved in the study
☒	☐ ChIP-seq
☒	☐ Flow cytometry
☒	☐ MRI-based neuroimaging

Antibodies

Antibodies used

Antibodies used were selected according to the antibody validation reported by the distributing companies (information is provided in the reporting summary). Mouse (IgG1) anti-MAP2 (Sigma-Aldrich; M4403; HM-2; 139117; 1:300), rabbit anti-PAX6 (Biolegend; 901301; B277104; 1:300), rat anti-BrdU (Abcam; ab6326; BU1/75 (ICR1); GR3365969-9; 1:300), rabbit anti-H3K27me3 (Cell Signaling Technology; 9733S; C36B11; 27; 1:300), rabbit anti-Ki67 (Invitrogen; MA-14520; SP6; SI2454941R; 1:300), rabbit anti-p21 Waf1/Cip1 (Cell Signaling Technology; 2947; 12D1; 12; 1:300), human anti-PAX6 (Miltenyi Biotec; 130-107-582; 5170301048); 1:300), mouse (IgG1) anti-Phospho-Histone H3 (Ser10) (Cell Signaling Technology; 9706S; 6G3; 10; 1:300), rabbit anti-SOX2 (Abcam; ab137385; GR3313268-18; 1:300), mouse (IgG2b) anti-TUBB3 (Sigma; T8660; SDL.3D10; 127270; 1:300).The following secondary antibodies were used (1:500 dilution): goat anti-mouse IgG1 Alexa 488 (Thermo Fisher; cat.no. A21121; 2083196), goat anti-mouse IgG Alexa 488 (Thermo Fisher; cat.no. A11001; 2140660), goat anti-rabbit Alexa 488 (Thermo Fisher; cat.no. A11008; 2521157), goat anti-rabbit Cy3 (Thermo Fisher; cat.no. A10520; 2160048), goat anti-rat Alexa 555 (Thermo Fisher; cat.no. A10522; 2153107), goat anti-mouse IgG1 Alexa 555 (Thermo Fisher; cat.no. A21127; 2110847)), goat anti-Human Alexa 555 (Thermo Fisher; cat.no. A21433; 2150293), goat anti-rat Alexa 633 (Thermo Fisher; cat.no. A21094; 2087716), goat anti-rabbit Alexa 633 (Thermo Fisher; cat.no. A21070; 2079350), goat anti-mouse IgG1 Alexa 633 (Thermo Fisher; cat.no. A21126; 2128996), Phalloidin-Atto647 (Sigma; cat.no. 65906).

Validation

Mouse (IgG1) anti-MAP2 (Immunohistochemistry (formalin-fixed, paraffin-embedded sections), Western Blot: using a fresh total rat brain extract or an enriched microtubule protein preparation); rabbit anti-PAX6 (Western Blot, Immunohistochemistry (Paraffin): Quality tested, Immunohistochemistry (Frozen): Reported in the literature, not verified in house); rat anti-BrdU (Flow Cytometry (Intra), Immunocytochemistry/Immunifluorescence, Immunohistochemistry (Paraffin)); rabbit anti-H3K27me3 (SimpleChIP® Enzymatic Chromatin IP Kits, CUT&RUN Assay Kit #86652, CUT&Tag Assay Kit #77552, Western Blot, Immunohistochemistry Leica Bond, Immunohistochemistry (Paraffin), Immunofluorescence (Immunocytochemistry), Flow Cytometry (Fixed/Permeabilized)); rabbit anti-Ki67 (Western Blot, Immunohistochemistry, Immunocytochemistry, Flow Cytometry); rabbit anti-p21 Waf1/Cip1 (Western Blot, Immunoprecipitation, Immunohistochemistry, Immunofluorescence, Flow Cytometry (Fixed/Permeabilized)), human anti-PAX6 (ICFC, 3D-Immunofluorescence, Immunofluorescence, Immunohistochemistry); mouse (IgG1) anti-Phospho-Histone H3 (Ser10) (Western Blot, Immunofluorescence (Frozen), Immunofluorescence (Immunocytochemistry), Flow Cytometry (Fixed/Permeabilized)); rabbit anti-SOX2 (Immunoprecipitation, Immunohistochemistry (Frozen, Paraffin), Immunocytochemistry/Immunifluorescence, Flow Cytometry, Western Blot); mouse (IgG2b) anti-TUBB3 (indirect ELISA, Western Blot: using a rat brain extract, Immunocytochemistry, Flow Cytometry)

Eukaryotic cell lines

Policy information about cell lines and Sex and Gender in Research

Cell line source(s)

HPSI0314i-hoik_1 (hoik1, hiPSC line, female, purchased at Wellcome Trust Sanger Institute, 77650129),

Authentication

Cell line purchased, not authenticated

Mycoplasma contamination

hoik1, A-ctrl, A-OS/het, A-OS/hom (MycoStrip50, Invivogen, rep-mysnc-50, 10619-46-02, 27.06.2024); hoik1, A-ctrl, C1-male, MID1-KO (LOOKOUT Mycoplasma PCR Detection Kit, Sigma, MP0035, BCCJ4343, 02.11.2023), J-ctrl, J-OS/het, J-OS/hom, C1-male, MID1-KO (LOOKOUT Mycoplasma PCR Detection Kit, Sigma, MP0035, BCCJ4343, 23.03.2023), hoik1, J-ctrl, J-OS/het, J-OS/hom (LOOKOUT Mycoplasma PCR Detection Kit, Sigma, MP0035, BCCD7621, 20.09.2021); M-ctrl, M-OS/het (LOOKOUT Mycoplasma PCR Detection Kit, Sigma, MP0035, BCCD7621, 19.05.2021); J-ctrl, J-OS/het, J-OS/hom, C1-male, MID1-KO, M-OS/male, M-OS/maleR (LOOKOUT Mycoplasma PCR Detection Kit, Sigma, MP0035, BCCC6349, 05.11.2020)

Commonly misidentified lines
(See ICLAC register)

Name any commonly misidentified cell lines used in the study and provide a rationale for their use.

Animals and other research organisms

Policy information about studies involving animals; ARRIVE guidelines recommended for reporting animal research, and Sex and Gender in Research

Laboratory animals	Pregnant C57BL/6 mice at embryonic day E13.5 were anaesthetized with isoflurane, and euthanized via cervical dislocation and embryos were harvested (E13.5).
Wild animals	Study did not involve wild animals.
Reporting on sex	Only cells of female embryos were ananlyzed. Sex was determined based on the detection of XIST.
Field-collected samples	Study did not involve samples collected from the field.
Ethics oversight	Animal experiments were conducted following German animal welfare legislation, and according to European (EU directive 2010/63/EU), national (TierSchG), and institutional guidelines.

Note that full information on the approval of the study protocol must also be provided in the manuscript.

Plants

Seed stocks	*Report on the source of all seed stocks or other plant material used. If applicable, state the seed stock centre and catalogue number. If plant specimens were collected from the field, describe the collection location, date and sampling procedures.*
Novel plant genotypes	*Describe the methods by which all novel plant genotypes were produced. This includes those generated by transgenic approaches, gene editing, chemical/radiation-based mutagenesis and hybridization. For transgenic lines, describe the transformation method, the number of independent lines analyzed and the generation upon which experiments were performed. For gene-edited lines, describe the editor used, the endogenous sequence targeted for editing, the targeting guide RNA sequence (if applicable) and how the editor was applied.*
Authentication	*Describe any authentication procedures for each seed stock used or novel genotype generated. Describe any experiments used to assess the effect of a mutation and, where applicable, how potential secondary effects (e.g. second site T-DNA insertions, mosiacism, off-target gene editing) were examined.*
